# A global poleward shift of atmospheric rivers

**DOI:** 10.1126/sciadv.adq0604

**Published:** 2024-10-11

**Authors:** Zhe Li, Qinghua Ding

**Affiliations:** Department of Geography and Earth Research Institute, University of California, Santa Barbara, Santa Barbara, CA, USA.

## Abstract

Atmospheric rivers (ARs) are key agents in distributing extratropical precipitation and transporting moisture poleward. Climate models forced by historical anthropogenic forcing suggest an increase in AR activity in the extratropics over the past four decades. However, reanalyses indicate a ~6° to 10° poleward shift of ARs during boreal winter in both hemispheres, featuring a rise along 50°N and 50°S and a decrease along 30°N and 30°S. Our analysis demonstrates that low-frequency sea surface temperature variability in the tropical eastern Pacific exhibits a cooling tendency since 2000 that plays a key role in driving this global AR shift, mostly over extratropical oceans, through a tropical-driven eddy-mean flow feedback. This mechanism also operates on interannual timescales, controlled by the El Niño–Southern Oscillation, and is less pronounced over the Southern Ocean due to weaker eddy activity during austral summer. These highlight the sensitivity of ARs to large-scale circulation changes driven by both internal variability and external forcing in current and upcoming decades.

## Teaser

The colder the tropical Pacific, the more atmospheric rivers shift toward higher latitudes globally.

## INTRODUCTION

Accelerating global warming in recent decades has contributed to a pronounced increase in the frequency and severity of extreme weather events globally, including heat waves (*1*–*3*), flash droughts (*4*, *5*), extreme precipitation (*6*–*9*), floods (*10*, *11*), and winter storms (*12*, *13*). More occurrences of such extremes regulate the hydrological cycle and result in an intensification of hydroclimate extremes and agricultural and ecosystem disasters (*14*–*17*). These changes pose diverse threats to human society and call for a closer examination of the underlying mechanisms driving these shifts in extreme precipitation and hydroclimatic patterns.

Anthropogenic forcing is widely recognized to induce a distinct poleward shift of certain extreme weather events, such as mid-latitude/extratropical storms (*18*–*25*) and tropical cyclones (*26*–*29*), in particular in the Southern Hemisphere (SH). Observational and modeling analyses have primarily attributed this shift to various mechanisms induced by global warming, including warmer sea surface temperature (SST) (*30*–*33*), and increased atmospheric water vapor (*20*, *34*) on a global scale; increased meridional temperature gradients (*18*, *35*, *36*), enhanced subtropical static stability (*32*, *37*, *38*), and stronger upper-level winds (*20*) over the subtropics; and higher tropopause height (*37*–*39*) and intensified troposphere heating (*40*) in the tropics. These mechanisms also collectively contribute to the expansion and weakening of the Hadley circulation (HC); thus, the poleward shifts of extreme weather events are viewed as a key signature of HC expansion in many studies focusing on hydroclimate response to global warming in the past and future (*26*–*29*, *38*). However, the exploration of the underlying mechanisms determining long-term changes in storms over the past decades is not yet thorough. This is because the variability of storms over the past decades was also strongly subject to tropical internal variability, such as the El Niño–Southern Oscillation (ENSO) and the Pacific Decadal Oscillation (PDO) (*38*, *41*, *42*), which can greatly regulate the location and strength of mid-latitude jets and the width of the HC. The brevity and uncertainty of observation records further hinder our ability to understand their past changes. These independent or interconnected dynamics, which explain changes in storms and originate from both anthropogenic and internal forcing, complicate attribution analyses, and emphasize the further need for a better understanding of the causal relationship between different climate drivers and responses in extreme weather events sensitive to extratropical storms.

Atmospheric rivers (ARs) are long, narrow corridors in the atmosphere characterized by strong horizontal water vapor transport (*43*, *44*). They can contribute to extreme weather events, especially when both strong extratropical storms and large-scale moisture supply are present. ARs have large impacts on extratropical weather extremes (*45*–*48*), mid-latitudes poleward moisture and energy transport (*43*), polar moistening (*49*–*51*), and various aspects of the extratropical hydrological cycle (*52*–*54*). Mirroring the observed poleward shifts in storm tracks documented by previous studies (*20*, *22*, *25*–*27*, *55*, *56*), a similar and concurrent poleward shift in ARs has also been increasingly discussed (*57*–*65*). Many of these studies focus on regional variability, such as in the North Atlantic, North Pacific, and the Southern Ocean. These studies primarily attribute the regional poleward shifts to the aforementioned poleward movement of the westerly jet induced by global warming (*57*–*59*, *66*), as well as other effects (*63*, *65*). However, the poleward shifts in ARs still remain a topic of debate, with some studies indicating an equatorward shift in the North Atlantic (*67*) and North Pacific (*66*) and others suggesting an opposite tendency (*57*–*65*). Furthermore, while some studies have demonstrated a global shift in AR tracks (*59*, *62*), the cause of these changes during the reanalysis era remains an open question. This knowledge gap makes it challenging to determine future changes of ARs and assess the reliability of climate models in replicating the similar feature.

Given that large-scale circulation changes over the past decades across different regions may be interconnected and share common underlying mechanisms, a comprehensive understanding of ARs’ responses to these circulation changes from a global perspective is thus crucial both in the recent past and a future warmer climate. This is particularly important when considering both anthropogenically- and naturally-driven factors that usually influence weather activity on a broader spatial scale (*65*, *67*, *68*). This global perspective is highly needed and essential for accurately predicting ARs and their associated hydrological extremes in the next decades to come. Especially, it has been well discussed that ARs will occur more frequently with higher intensity and heavier precipitation in a warming climate due to increased availability of water vapor and stronger dry static stability in the atmosphere (*57*, *66*, *69*–*72*). Nevertheless, whether these existing theories, which emphasize enhanced moisture concentration and static stability in the atmosphere, as well as a poleward shift in the westerly jet and storms, can adequately explain all key features of the observed global AR changes and further aid us in projecting ARs in the future remains unanswered, as the complexity of AR dynamics is yet to be fully understood.

In this study, we examine recent global-scale changes in ARs over the past four decades and their roles in shaping climate-weather interactions around mid-latitudes. In reanalysis, we find that AR frequency trends exhibit a poleward shift globally, stemming from a tropical origin, which is not well captured by the ensemble mean of a climate model forced by anthropogenic forcing. To understand this discrepancy, we use a fingerprint pattern matching analysis, emphasizing simulations by individual members in the ensemble. Our results reveal that the poleward shifts of ARs are closely associated with a tropical-driven eddy-mean flow feedback, and simulations reflecting the model’s internal variability can better capture the observed tropical-circulation-AR connection than the ensemble mean. The expected outcome of this study is to tease apart the relative roles of anthropogenic and natural forcing in shaping the observed changes of ARs over the past decades with greater confidence and to understand how this finding can aid us in improving future global-scale projections of ARs.

## RESULTS

### Observed and simulated poleward shifts of ARs

In the past few decades, the long-term trends of AR frequency have exhibited a clear well-organized global pattern in boreal winter, while showing more complex regional variability in other seasons (fig. S1). Thus, this study mainly focuses on understanding the global long-term changes and underlying mechanisms of ARs in December-January-February (DJF). We also find that the mechanism driving the global AR frequency trend pattern in DJF may exert similar impacts in March-April-May (MAM), which is briefly discussed in the last section. Notably, in the SH, the trends of AR frequency appear similar across all seasons, whereas in the North Hemisphere (NH), the annual mean trend more closely resembles the trends observed in DJF and MAM, indicating the dominance of these two seasons in shaping annual mean variability.

**Fig. 1.**
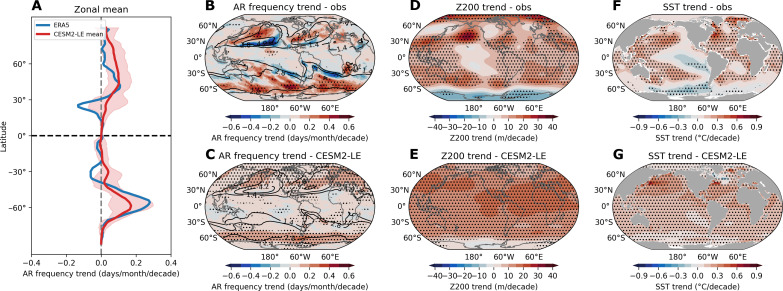
Observed and simulated historical trends of AR frequency, circulation, and SST. (**A**) The zonal mean of December-January-February (DJF) atmospheric river (AR) frequency trends from ERA5 (blue line) and CESM2-LE ensemble mean (red line) for the historical period 1979–2022. The red shading in (A) indicates the fifth and 95th percentile of the CESM2-LE members. (**B** and **C**) The linear trends of DJF AR frequency from ERA5 (B) and CESM2-LE ensemble mean (C) for the historical period 1979–2022. (**D** and **E**) Same as (B) and (C) but for DJF geopotential height at 200 hPa (Z200) from ERA5 (D) and CESM2-LE ensemble mean (E). (**F** and **G**) Same as (B) and (C) but for DJF sea surface temperature (SST) from ERSSTv5 (F) and CESM2-LE ensemble mean (G). Black stippling in all plots indicates statistically significant trends at the 95% confidence level.

An increase in DJF AR frequency is particularly significant along the 50° to 60° latitudes in both hemispheres (up to 0.59 days/month/decade), including the North Atlantic and Pacific Oceans, as well as the entire Southern Ocean (Fig. 1, A and B). In contrast, the subtropics along the 30° latitudes of both hemispheres have experienced a decrease in DJF AR frequency. The larger increase in AR frequency occurs on the poleward flank of its climatological peak region, while the strong decrease is observed within this peak region, most notably in the North Pacific (Fig. 1B). This dipole pattern suggests a global-scale poleward shift of active DJF AR frequency regimes by approximately 10° over recent four decades. Correspondingly, positive trends in AR frequency over the extratropics have led to similar trends in total precipitation and northward moisture transport during the same period, in particular over the North Pacific (fig. S2). This strong increase in AR frequency in the North Pacific may lead to heightened precipitation and transports more moisture poleward, potentially extending into the Arctic region through the Bering Sea (around 180°E). Concurrently, DJF geopotential height at 200 hPa (Z200) rises mostly over the extratropics in both hemispheres, exhibiting a wave train structure along the subtropics, with successive isolated barotropic high-pressure centers emerging over the North Pacific (Fig. 1D). The polar regions also display a contrasting polarity, with a Z200 rise in the Arctic and a decrease in the Antarctic (Fig. 1D), possibly due to Arctic Amplification and the ozone depleting effect in their respective regions. DJF SST trends exhibit significant warming almost everywhere, except for slight cooling of around −0.2°C per decade in the eastern Pacific and Southern Oceans (Fig. 1F).

Compared to observed AR activity in ERA5 (Fig. 1B), the Community Earth System Model v2 Large Ensemble (CESM2-LE) shows a more moderate increasing trend (up to 0.24 days/month/decade) in AR frequency at high latitudes in both hemispheres (Fig. 1C). While the ensemble mean from CESM2-LE, representing forced trends driven by anthropogenic forcing, fails to capture the significant negative trend signals observed in the subtropics along 30°N and 30°S, some individual ensemble members do better in reflecting this change (Fig. 1, A and C). This discrepancy between the reanalysis and simulation is also evident in the area-weighted global average DJF AR frequency, where CESM2-LE favors an overall increasing trend, while ERA5 shows notable interdecadal variability with a decreasing trend from 1979 to 2013, followed by an abrupt increase in recent 10 years (fig. S3A). ERA5 indicates a decreasing trend in tropical average AR frequency, while CESM2-LE shows no trend (fig. S3B); yet both exhibit an increasing trend in extratropical AR frequency (fig. S3C).

Moreover, the observed latitude of maximum zonally averaged AR frequency shifts poleward by about 6° in the NH (last 5 years mean, 33°; first 5 years, 27°) and about 10° in the SH (last 5 years mean, −51°; first 5 years, −40.5°) over the past four decades, while CESM2-LE mean shows nearly no shift in the NH and about 4° in the SH (last 5 years mean, −48°; first 5 years, −44°) (fig. S4, A and C). This shift in ERA5 also features an apparent poleward movement of the core AR frequency region (>4.2 days/month, representing the 85th percentile of 44-year DJF AR frequency in ERA5) in recent decades in the SH and North Pacific, and a significant expansion of this core region in recent years, nearly doubling the core domain compared to the 1980s (fig. S4, B and D). In contrast, CESM2-LE shows a contrasting pattern with only a slight expansion of the core AR region in the North Atlantic and Southern Oceans and no apparent poleward shift. Overall, the forced response in CESM2 features a rise of ARs in the high latitudes without a decrease in the lower latitudes, differing from the observed poleward shifts characterized by opposite-sign changes between 30°N and 50°N (or 30°S and 50°S). The differences between the reanalysis and CESM2-LE, along with the spread among model members, suggest that additional factors beyond global warming play an important role in driving the observed global poleward shifts of ARs.

Similarly, the comparison of Z200 between ERA5 and CESM2-LE reveals distinct patterns: CESM2-LE ensemble mean shows a rather uniform global increase (Fig. 1E), while ERA5 displays spatial heterogeneities with stronger increasing trends in the subtropics, weaker trends in the tropics, and decreasing trends in the tropical Pacific (Fig. 1D). This observed large-scale circulation trend in ERA5 features a clear cold SST anomaly-driven Gill pattern response (a pair of symmetric upper tropospheric low-pressure systems straddling the equator) in the tropical Pacific (fig. S5) and an apparent wavy structure in the extratropics (Fig. 1, D and F). However, CESM2-LE does not replicate this negative diabatic heating-driven response in the tropics and the wavy structure of large-scale circulation outside the tropics (Fig. 1, E and G). The pattern of AR frequency trend in ERA5 shows a notable similarity to the observed changes in large-scale circulation in ERA5 and SST in Extended Reconstructed Sea Surface Temperature, version 5 (ERSSTv5), indicating the importance of circulation variability in modulating global decadal variability of ARs, potentially due to the impact of anomalous diabatic heating shaped by tropical Pacific SST cooling anomalies. These indicate that more attention should be devoted to better understand the relationship between large-scale circulation and ARs, which is key to identifying why the model may fall short in accurately replicating observed AR frequency trends (Fig. 1B).

### Statistical relationship between atmospheric circulation and AR frequency

**Fig. 2.**
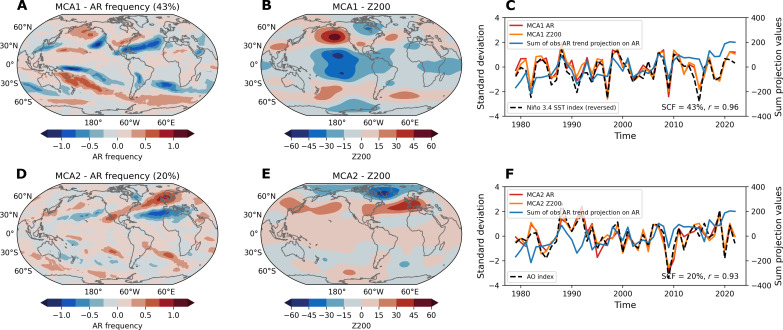
Observed statistical relationship between detrended AR frequency and circulation. (**A** to **C**) Results of the leading maximum covariance analysis mode (MCA1) of detrended global DJF AR frequency and geopotential height at 200 hPa (Z200) from ERA5 for the historical period 1979–2022, with spatial patterns of AR frequency (A) and Z200 (B), and their corresponding standardized time series (C). The time series of the sum of raw AR frequency projection on long-term AR frequency trend from ERA5 (blue) and reversed DJF Niño 3.4 SST index from ERSSTv5 (black dashed) are shown in (C). (**D** to **F**) Same as (A) to (C) but for results of the second MCA mode (MCA2) of two variables. The NOAA DJF Arctic Oscillation (AO) index is shown in (F). “SCF” in (C) and (F) indicates the squared covariance fraction of the MCA mode, and “*r*” in (C) and (F) indicates the correlation coefficient between the MCA mode time series. The linear trends of DJF AR frequency and Z200 are removed (detrended) for the MCA.

To statistically investigate the coherent patterns between large-scale atmospheric circulation and ARs, we apply the maximum covariance analysis (MCA) method to detrended Z200 and AR frequency from 1979 to 2022 during boreal winter (see Materials and Methods). The first MCA mode (MCA1) accounts for 43% of the covariance and is shown in Fig. 2 (A to C). The spatial pattern of AR frequency in MCA1 predominantly shows positive signals in the extratropics, in particular over the North Pacific, and a tilted positive band extending from the tropics to the subtropics in the Southern Ocean (Fig. 2A). This pattern largely resembles the observed DJF AR frequency trend (Fig. 1B), although it lacks some positive signals in the Southern Ocean. The MCA1 Z200 pattern displays an ENSO-driven Pacific-North American (PNA) and Pacific-South America (PSA) global teleconnection pattern, with a Gill response over the eastern tropical Pacific (Fig. 2, B and C). This Z200-AR mode is highly correlated to typical ENSO-related SST anomalies in DJF, marked by negative signals in the eastern Pacific Ocean and positive signals in the subtropics on both sides of the Pacific Ocean (fig. S6A). This correlation pattern aligns with the observed long-term trend of DJF SST, especially in the Pacific Ocean (Fig. 1F), suggesting that the long-term trends in Z200, SST, and AR are closely interconnected, and their connections may stem from the ENSO-driven teleconnection reflected by MCA1. The SST cooling in the eastern Pacific Ocean may represent a more frequent occurrence of La Niña events (*73*) in recent decades, especially since 2000, which have an imprint on low-frequency SST variability in the region.

The second MCA mode (MCA2) explains 20% of the covariance and its spatial patterns feature a coupling of the two fields in and around the Arctic. The AR frequency pattern in MCA2 is characterized by positive signals in the North Atlantic and North Pacific and negative signals along 30° (Fig. 2D). This pattern contributes to the observed increasing trend in AR frequency in the North Atlantic and North Pacific, which is less captured in the MCA1 results. MCA2 Z200 is dominated by a prominent Arctic Oscillation variability (Fig. 2F), with negative signals above Greenland and positive signals in the NH subtropics (Fig. 2E), and is highly related to cooling SST in the subpolar North Atlantic (fig. S6B). When this mode is in its positive phase, a strong mid-latitude jet stream with stronger westerlies steers ARs northward, while in its negative phase, a weaker and more meandering jet dips farther south, causing ARs to shift equatorward.

Moreover, the MCA over the past 44 years using raw data shows results similar to those of the detrended MCA (fig. S7). It suggests that the Z200-AR connection is robust on both interannual and interdecadal timescales over the past decades, and the observed long-term changes of Z200 and AR frequency may reflect a low frequency variation of the interannual AR-Z200 connection, as shown in Fig. 2 (A and B).

To further quantify which MCA AR patterns mostly capture the observed long-term trends of ARs, we project the DJF AR frequency anomalies for each year onto the spatial pattern of long-term DJF AR frequency trend. Through this calculation, a 44-year time series can be obtained by globally summing the projected spatial values in each DJF. By design, this time series (the blue curve in Fig. 2, C and F) displays a clear upward trend over the period, indicating a gradual enhancement of the AR trend signal over time. The correlations of this projected time series with MCA1&2 AR time series inform us which MCA mode is more critical in explaining the overall AR variability on a global scale. This constructed time series exhibits a significant correlation with the detrended MCA1 AR time series (Fig. 2C), with a correlation coefficient of 0.73 (detrended) on interannual timescales. This indicates that the majority of the observed long-term changes in ARs can be well explained by the MCA1 AR-Z200 coupling mode. Although the time series of MCA2 AR shows a weaker association (*r* = 0.17, detrended) with the observed AR trend projection time series (Fig. 2F), this mode still accounts for some aspects of AR changes in the North Atlantic and North Pacific. If we redo this calculation by focusing on the NH, MCA2 appears to be important in capturing the observed AR trends in the NH (*r* = 0.48, detrended).

### Contribution of eddy-mean flow interactions to poleward shifts in ARs

The underlying reason for a zonal-oriented height rise along the NH and SH subtropics, in tandem with tropical SST cooling in the eastern tropical Pacific over the past four decades, remains to be fully explored (Fig. 1, D and F). The eddy-mean flow feedback usually plays a key role in contributing to striking zonal mean anomalies in large-scale circulation (see Materials and Methods). In the following analysis, we mainly consider zonal mean changes of zonal winds during a quasi-equilibrium state [($\partial \bar{u}/\partial t$) ~ 0] in the upper troposphere [($\alpha \bar{u}$) ~ 0]. The feedback occurs when the divergence of northward eddy momentum fluxes [∂(*u*′*v*′)/∂y] is nonzero over some latitudes, so the only term to balance it is zonal mean of meridional wind ($\bar{v}$), which will eventually alter the intensity of the zonal mean of zonal flow ($\bar{u}$) through some adiabatic processes and the thermal wind relationship. Once the zonal mean of zonal winds vary, it is able to further modify the eddies through modifying their related baroclinicity and preferred wavelength. These interactions create a feedback scenario that increases the intensity and persistence of the zonal mean component of anomalous large-scale atmospheric circulation (*74*–*76*). Given the important role of eddy-mean flow feedback in regulating zonal mean flow variability (*77*, *78*), we place more attention on its role in maintaining the long-term Z200 trend in this section.

**Fig. 3.**
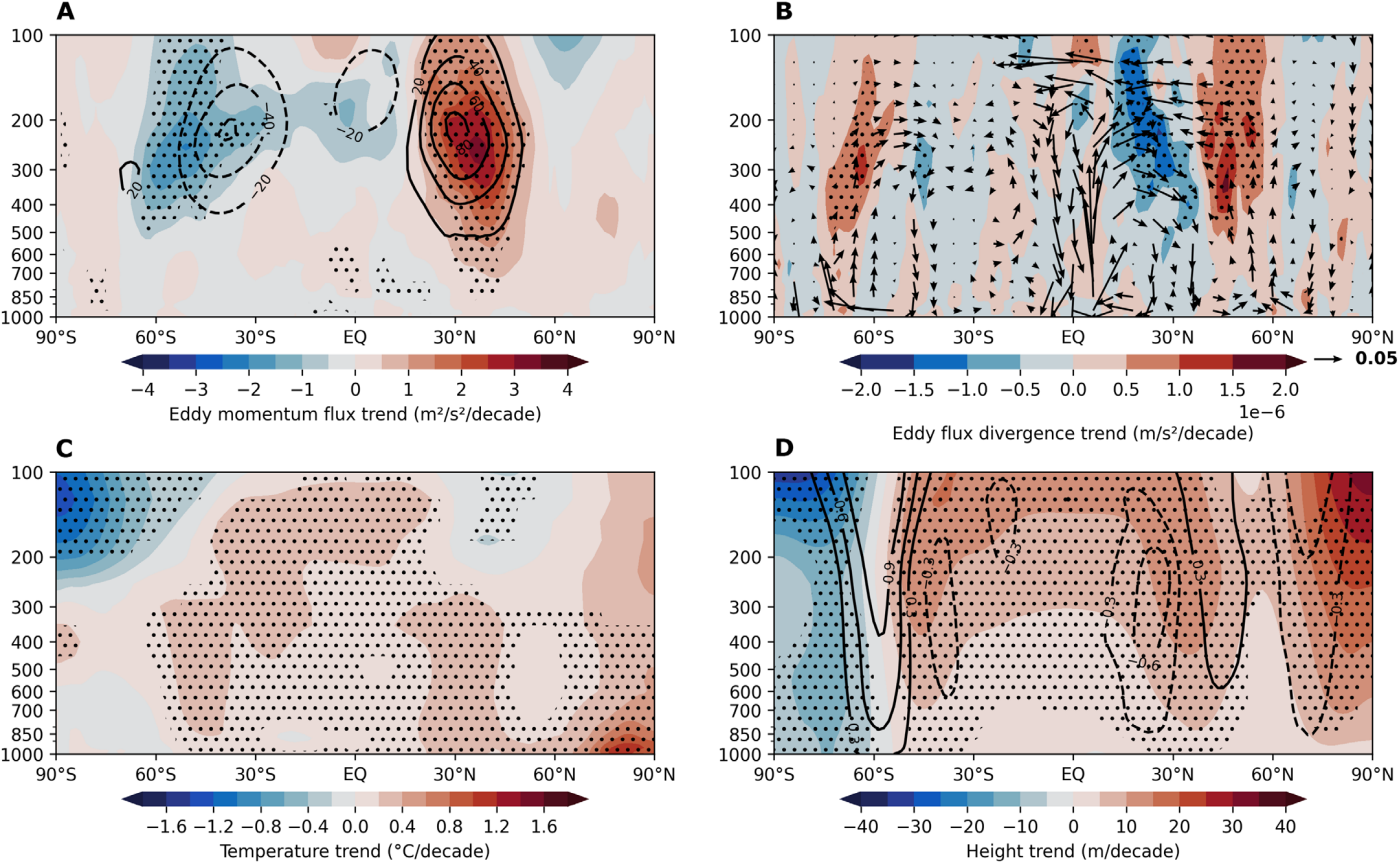
Observed trends in zonal averaged eddy momentum flux, circulation, temperature, and winds. (**A**) The linear trend (shading) and 44-year mean (contour) of DJF zonal averaged northward eddy momentum flux ($\bar{u\prime v\prime }$). (**B**) The linear trends of convergence/divergence of DJF zonal averaged northward eddy momentum flux ($\partial \left(\bar{u\prime v\prime }\right)/\partial y$, shading) and DJF zonal averaged meridional overturning circulation [cross section: vertical velocity (ω⋅−50; Pa/s) and meridional velocity (m/s), vectors]. (**C**) The liner trend of DJF zonal averaged air temperature. (**D**) The linear trends of DJF zonal averaged geopotential height (shading) and zonal winds (contour). All calculations are based on the historical period 1979–2022 from ERA5. Black stippling in all plots indicates statistically significant trends at the 95% confidence level.

To better illustrate this idea, we calculate the past 44-year long-term trends of DJF northward eddy momentum flux and its meridional convergence/divergence (see Materials and Methods). It is clearly seen that the significant trend of northward eddy momentum flux aligns well with the height rise over both subtropics, which also shifts slightly poleward to the maximum action center of the climatological eddy momentum fluxes (Fig. 3, A and D). The strengthened divergence and convergence of eddy momentum flux appear around 25°N and 45°N, respectively, which favor enhanced zonal mean southerly and northly winds over the same regions (Fig. 3B). These winds converge over ~35°N and consequently induce a sinking motion and adiabatic warming from the surface to ~300 hPa (Fig. 3, B and C). Thus, the entire air column around 30° to 40°N is adiabatically warmed and experiences a rise in the height field with the maximum rise at ~300 hPa. A similar pattern occurs over the SH subtropics around 40° to 50°S, but with slightly weaker intensity, probably due to reduced climatological eddy activity during this season (Fig. 3A).

**Fig. 4.**
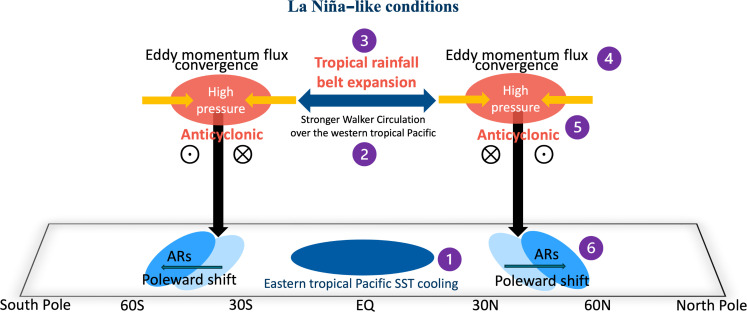
A schematic diagram illustrating the mechanism explaining the global shifts of ARs. The functioning of this mechanism can be viewed as a progression of sequenctial processes from step 1 to step 6. During the La Niña conditions (or a long-term SST cooing mirroring the La Niña conditions, step 1), SST cooling over the eastern tropical Pacific triggers a stronger Walker circulation over the western tropical Pacific (step 2), which favors an expansion of the tropical rainfall belt (step 3). This tropical expansion, along with the eddy momentum flux (step 4), will further induce the high-pressure anomalies with anticyclonic wind anomalies (step 5), resulting in a poleward shift pattern of AR frequency via the steering effect (step 6). This shift is characterized by an increase in AR frequency on the poleward side of the subtropics and a decrease on the tropical flank. Conversely, during the El Niño conditions (or a long-term SST warming in the tropical eastern Pacific), the mechanism operates in the opposite direction. See the main text for a more detailed description of the mechanism. The blue double-headed arrow indicates the tropical rainfall belt expansion. The yellow and black arrows indicate the zonal mean overturning circulation induced by the convergence of eddy momentum flux around the extratropics.

This is consistent with the well-established mechanism explaining the tandem changes of tropical SST forcing and height rise in the extratropics (*79*–*84*). To make progress, we develop this mechanism to highlight its impacts on ARs on low-frequency timescales. As summarized in our schematic diagram (Fig. 4), this line of thought suggests that eastern tropical Pacific SST cooling, similar to the La Niña conditions or the negative phase of the PDO, favors an expansion of the tropical zone due to stronger tropical waves arising from a stronger Walker circulation over the western tropical Pacific. This tropical expansion can be inferred from the trend of tropical precipitation over the past four decades (fig. S8). It is known that during La Niña winters, precipitation along the equator becomes weaker but strengthens along 15°N and 15°S compared to El Niño years (fig. S8, C and D). This suggests that the total amount of tropical rainfall may not differ significantly between the two conditions but is redistributed within the tropics, with more expansion toward the subtropics in La Niña years. The long-term trends of precipitation resemble this El Niño to La Niña expansion (fig. S8B), likely due to a trend of more La Niña-like conditions in the tropical Pacific since 2000. In the mid-latitudes, this expansion effect alters the features of subtropical storms and diverts them from their usual action zones due to various mechanisms (*41*, *85*, *86*). The shifts in storm tracks modify the zonal mean eddy momentum flux as shown in Figs. 3 and 4 and induce high-pressure anomalies along both subtropics.

These high-pressure anomalies, in particular over the extratropical oceans with anticyclonic wind anomalies, generate strong westerlies on their poleward side and easterlies on the tropical flank. These zonal wind anomalies induce the shifts of ARs, leading to an increase in AR frequency toward higher latitudes and a decrease nears the equatorward side of the subtropics (fig. S9 and Fig. 4). Although ENSO-related wave trains on both hemispheres (PNA and PSA) may imprint some zonal mean signals along the subtropics, the main component of the zonal mean pattern is believed to be driven by this eddy-mean flow feedback, which plays an important role in linking tropical forcing to the poleward shifts of ARs.

### Fingerprint analysis detecting roles of circulation and tropical SST in regulating ARs

Considering the inferred role of tropical SST forcing in driving ARs activity via the eddy-mean flow feedback, as demonstrated in the previous sections, we expect that simulations with observed tropical SST variations imposed should more accurately replicate observed AR trends than those without such tropical forcing added. To test this hypothesis, we use 40 ensemble members from CESM2-LE, which provide 6-hourly data for AR detection (see Materials and Methods). These members are differentiated by their initial conditions, allowing us to access the different AR behaviors among members and the extent to which they are affected by the aforementioned tropical forcing.

Although the ensemble mean of CESM2-LE does not fully capture the observed poleward shifts of ARs, certain individual members exhibit patterns that resemble the observed trends to some level (fig. S10). The difference among these members suggests that internal variability can partially contribute to the observed poleward shifts of ARs over the past decades. To better reveal the role of internal variability (or tropical SST forcing) in the observed changes of ARs, we apply a fingerprint pattern matching analysis using the 40 members of CESM2-LE (see Materials and Methods). We identify two groups performing very differently in simulating the observed long-term trends of DJF AR frequency over the same period: a “top group” comprising six members that show the most positive spatial correlations with the observed DJF AR frequency trend (see Materials and Methods, beyond the 85th percentile of the ascending sorted spatial correlations), and a “bottom group” comprising six members with the most negative spatial correlations (or near zero, below the 15th percentile).

**Fig. 5.**
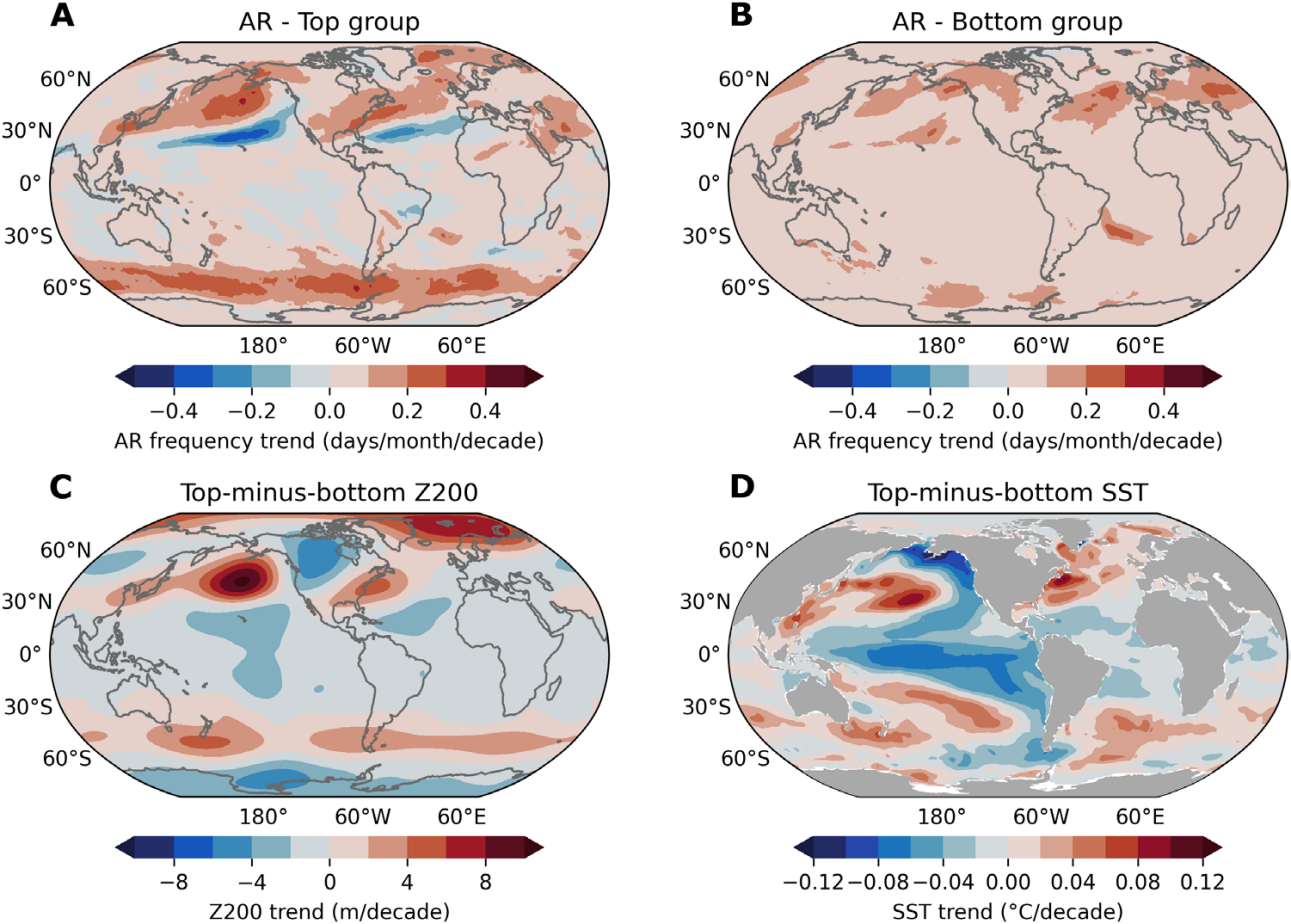
Differences in simulated AR frequency trends among CESM2-LE 40 members. (**A** and **B**) The linear trends of DJF AR frequency from the ensemble average of six members in CESM2-LE 40 members that show the greatest similarity to the observed trend pattern of DJF AR frequency [(A), top group)] and the ensemble average of six members that show the weakest similarity to the observed trend pattern of DJF AR frequency [(B), bottom group)] for the historical period 1979–2022. (**C** and **D**) The difference of ensemble averaged DJF geopotential height at 200 hPa (Z200) (C) and SST (D) trends between the top and bottom groups for the historical period 1979–2022. The difference is divided by 2 to reflect the member-spread in one direction.

By design, the DJF AR frequency trend in the top group ensemble features a similar pattern to the observed changes, although it lacks significant trends of AR in the SH subtropics (Fig. 5A), indicating that when member-to-member diversity is considered, some members can still capture the poleward shifts of ARs. In contrast, the bottom group shows sporadic positive trends in the subtropics rather than the observed well-organized positive-negative contrasting pattern (Fig. 5B). The differences in Z200 and SST trends between these two composites (hereafter “top-minus-bottom”) are shown in Fig. 5 (C and D). Since both groups are subject to the same anthropogenic forcing, their differences minimize the impact of global warming and mainly represent the influence of internal variability. The most prominent feature in the top-minus-bottom Z200 composite is an isolated high pressure in the North Pacific, with a high-pressure band in the Southern Ocean and lower pressure in the tropical Pacific (Fig. 5C). This pattern bears a resemblance to the observed DJF Z200 trends (Fig. 1D), including the contrasting trends between the two polar regions. Similarly, the top-minus-bottom SST composite (Fig. 5D) favors a similar cooling pattern in the tropical Pacific as observed in ERSSTv5 (Fig. 1F) and is consistent with the MCA results (Fig. 2 and fig. S6A). These results suggest that in the model’s world, the observed AR trends are better simulated by certain members that also better replicate observed large-scale circulation and tropical SST variability. This indicates a strong physical constraint of tropical-driven large-scale circulation in shaping the long-term trend of global ARs, independent of anthropogenic forcing.

**Fig. 6.**
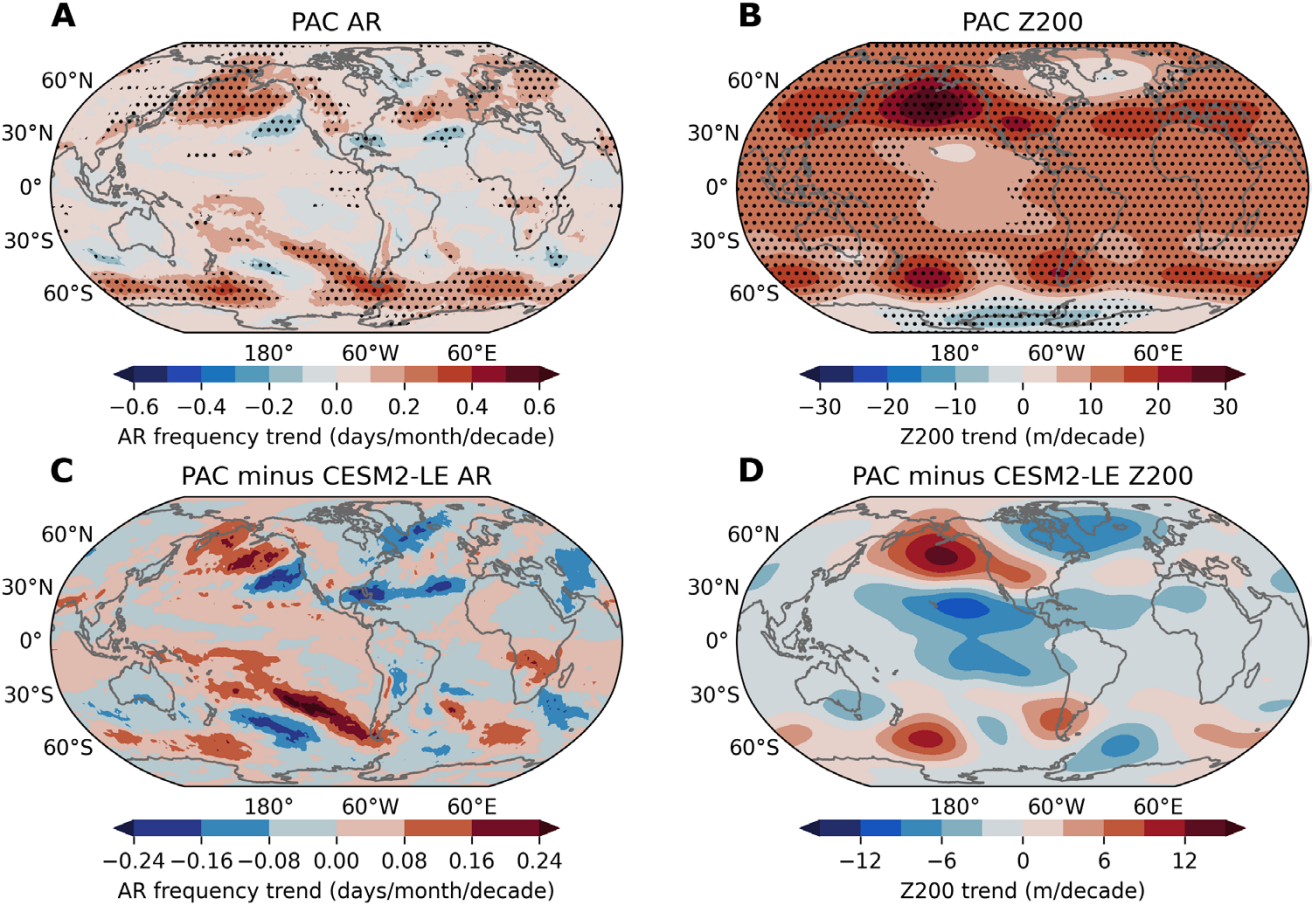
Simulated trends in AR frequency and circulation in PAC simulations. (**A** and **B**) The linear trends of DJF AR frequency (A) and geopotential height at 200 hPa (Z200) (B) from the ensemble mean of PAC simulations for the historical period 1979–2018. (**C** and **D**) The difference of the linear trends of DJF AR frequency (C) and Z200 (D) between the ensemble mean of PAC and CESM2-LE for the historical period 1979–2018. Black stippling in all plots indicates statistically significant trends at the 95% confidence level.

We further examine the pacemaker experiment (PAC), a 10-member ensemble from CESM2, wherein SSTs in the eastern tropical Pacific are nudged to time-varying prescribed SST anomalies derived from ERSSTv5, while remaining fully coupled everywhere else (see Materials and Methods). Meanwhile, radiative and anthropogenic forcing is imposed in the PAC ensemble. Thus, the results from PAC reflect CESM2’s response to both anthropogenic forcing and observed SST variability originating from the eastern tropical Pacific. DJF AR frequency trends in the PAC ensemble exhibit a poleward shift pattern as observed, with strong positive trends occurring over the North Pacific and extending from the tropics to the subtropics in the Southern Ocean, alongside negative trends in the NH subtropics (Fig. 6A). Similarly, DJF Z200 in the PAC ensemble shows an observation-like pattern, with negligible increasing trends over the tropical Pacific compared to other regions and the two belts of high-pressure wavy activity over the subtropics (Fig. 6B).

Since CESM2-LE is forced by the same anthropogenic forcing but without observed tropical Pacific SST forcing, differences between PAC and CESM2-LE simulations may provide insights into CESM2’s response solely to observed eastern tropical Pacific SST forcing. The difference in AR frequency trends between PAC and CESM2-LE shows a clear opposite-sign dipole pattern, especially in the NH (Fig. 6C), which suggests that the model with imposed tropical Pacific SST forcing effectively captures the observed AR changes there. Moreover, the difference in Z200 trends between PAC and CESM2-LE successfully replicates the spatial structure of the observed Z200 changes, with significant negative trends in the tropical Pacific and high-pressure belts along the subtropics, particularly over the North Pacific (Fig. 6D). However, it is noted that the decreasing trends in both hemispheres in AR responses in Fig. 6A are weaker than those observed (Fig. 1B), and some Z200 responses in Fig. 6B still differ from those in the fingerprint analysis (Fig. 5C) and ERA5 (Fig. 1D) over parts of the North Atlantic and near West Antarctic. These discrepancies indicate that 10 members of PAC may not sufficiently reflect a robust response to tropical SST forcing in the model. This inability may also imprint its biased signal on ARs in those regions. Even so, these results reinforce the importance of eastern tropical Pacific SST cooling in regulating long-term AR changes with a poleward shift pattern through altering large-scale circulation patterns in most areas of the globe, and this SST-Z200-AR coupling is partially driven by internal variability.

## DISCUSSION

In this study, we show low-frequency variability of ENSO strengthens subtropical high pressures on both sides of the tropics, exerting a strong impact on an intensification of boreal winter AR frequency in the mid-latitudes along 50° to 60°N and 50° to 60°S and a diminish of AR frequency around 30°N and 30°S. A tropical eddy-mean flow feedback acts as an important mechanism to maintain the strengthening of the high-pressure anomalies and the resultant poleward shift pattern of ARs. Using state-of-the-art model ensemble and a fingerprint pattern matching method, we further suggest that global climate model simulations may better capture observed AR changes when observed low-frequency SST variability is well simulated, raising a need to close examine their future projections in the tropics and related teleconnections and eddy-mean flow feedback to the extratropics. Thus, our study provides an improved physical understanding of the mechanisms driving the recent global poleward shifts of ARs, which can further assist in assessing future projections of weather extremes in a warmer climate.

While the currently widely accepted view emphasizes on a thermodynamic control of global warming on extreme precipitation via various mechanisms (*87*, *88*), our findings suggest that tropical-driven circulation changes, often underestimated by climate models and recognized as a key source of uncertainty (*59*), may also play an important role in extreme precipitation in the extratropics through regulating the westerly jets and AR activity. This dynamic component is critical for accurately predicting future extreme weather patterns and may either mask or amplify the signals due to global warming, depending on the phase of the low-frequency component of ENSO. Thus, to improve the capability of climate models in projecting future climate extremes, especially in the extratropics, we should ensure that climate models own a reasonable skill in replicating the observed tropical-circulation-AR connection and low-frequency ENSO variability.

Considering the importance of tropical SST variability in shaping global DJF AR changes over the past decades, it is plausible that future changes in tropical SST may still hold some potential to modulate ARs through the identified eddy-mean flow feedback mechanism. Comparing the final 40 years of CESM2-LE projections (2060–2099) under the Shared Socioeconomic Pathway (SSP) 3-7.0 scenario with the recent four decades reveals a noticeable increase in DJF AR occurrences, extending into mid-latitudes and the Arctic, with frequencies potentially doubling by the century’s end, in particular in the SH (fig. S11, A and C). This projected increase in ARs is expected to induce more extreme precipitation events in the extratropics. In addition, it is noted that future AR changes exhibit a slight decline in the subtropics, although this decline is much weaker than the trends observed over the past four decades. This suggests that even under significant global warming (SSP3-7.0) forcing in CESM2, the climate response in the subtropics does not favor a significant long-term decrease in ARs there. This is likely because an increase in moisture supply and a northward shift of storms under global warming have contrasting impacts on the generation of ARs around the subtropics, leading to minimal changes there. However, the ultimate cause of this feature remains unresolved. Regarding seasonality, future projections for the SH reveal similar trends for JJA and DJF, while in the NH, there is a significant difference between the two seasons. JJA projections indicate a more pronounced increase in AR activity in the Arctic, whereas DJF projections exhibit an increase in the extratropics (fig. S11, A and B). This discrepancy suggests a seasonal variation in AR behavior and is likely due to the most pronounced Arctic Amplification in summer. Despite these differences, both seasons demonstrate extensive penetration of ARs into the Arctic, which could greatly influence Arctic moisture concentration and consequent impact on the cryosphere.

Since ENSO, usually peaking in DJF, may preferentially exhibit long-term variations during its peak season, it is understandable that the mechanisms identified in this study are most pronounced in boreal winter. To further assess whether the same mechanisms also operate at other times of the year, we extend our investigation to other seasons and find that a similar connection exists in MAM. However, during boreal summer and fall, when ENSO is relatively weak, the identified mechanisms seem to be greatly weakened in their global impact. This weakening may be due to changes in the extratropical basic state that do not favor a strong eddy-mean flow feedback or tropical-extratropical teleconnections emanated from the ENSO region. However, we cannot rule out the possibility that in a warmer world, the seasonal dependence of these impacts may vary, which deserves further attention.

Moreover, some recent studies have suggested the important role of aerosol forcing in contributing to the recent tropical Pacific SST cooling since the 1980s (*89*) and in weakening ARs (*68*), implying that the mechanisms discussed in this study might be anthropogenically driven by aerosol-induced radiative forcing in the tropics. However, although aerosol forcing is well considered and specified in CESM2-LE, the SST cooling trend pattern suggested by the study (*89*) is not clearly seen in the ensemble mean response in our analysis. This suggests that it is still premature to attribute the observed SST cooling over the eastern Pacific and related impacts on ARs solely to an anthropogenic origin. It calls for a more thorough future analysis to understand whether the SST cooling pattern is driven by internal processes or external forcing, which may also represent an important source of uncertainty in attributing observed tropical and extratropical variabilities and in projecting future changes in tropical SST and their related impacts on extratropical ARs.

Our findings have important and broader implications beyond the climate research community. The global shifts of ARs may alter established precipitation patterns, affecting regional water resources by potentially reducing water availability in areas that historically rely on AR-driven precipitation while increasing flood risks in newly affected regions. The increase in AR frequency in higher latitudes could lead to more frequent and intense extreme weather events, such as severe storms and heavy rainfall, posing challenges to climate prediction efforts for these areas. In contrast, regions experiencing fewer ARs might face prolonged heat waves or droughts, worsening water shortages and climate stress for local ecosystems. Considering the prominent shifts of ARs over the extratropical ocean, how the ocean responds to these AR-induced changes requires further attention. In addition, our emphasis on the tropical-extratropical linkages and climate-extreme interactions highlights the importance of improving projections of ENSO variability and related eddy-mean flow feedback to enhance predictions of extratropical circulation and AR activity in the next decade to come. This could provide a roadmap for refining climate projections under continuous influences of both anthropogenic forcing and natural variability, aiding in the development of more effective adaptation and mitigation strategies.

## MATERIALS AND METHODS

### Reanalysis and observation data

The ERA5 reanalysis (*90*) is used in this study for atmospheric variables, including geopotential height, horizontal winds (meridional and zonal wind velocities), omega (vertical velocity), air temperature, and total precipitation spanning the historical period from 1979 to 2022 during the boreal winter (DJF). Geopotential height at 200 hPa (Z200) is used in study because, at this level, our physical interpretation centering on tropical-extratropical teleconnections and the eddy-mean flow feedback is less influenced by frictional and topographic effects. Six-hourly variables are used for the AR detection algorithm; otherwise, monthly variables are used for other analyses. Monthly means of the vertical integral (from the surface to the top of the atmosphere) of northward water vapor flux (*91*) are derived from 1-hourly ERA5 reanalysis. Winds used for computation of water vapor flux are mass-adjusted on the basis of the diagnosed imbalance between divergence of vertically integrated dry mass flux and tendency of dry air mass (*91*).

SSTs are obtained from the National Oceanic and Atmospheric Administration (NOAA) ERSSTv5 (*92*) covering the same period and seasons. The Niño 3.4 SST index, averaging SST anomalies over the region 5°N to 5°S and 170° to 120°W, is calculated to reflect ENSO’s variations. An observation-based precipitation product, Global Precipitation Climatology Project version 2.3 (*93*), is also used in this study to study tropical precipitation changes.

### Model experiments

We analyze the same variables as those in the reanalysis using the CESM2-LE (*94*) 40 members for both historical (1979–2022) and future simulations extending to 2100. These simulations are forced by Coupled Model Intercomparison Project Phase 6 (CMIP6) historical forcing from 1850 to 2014, followed by the SSP3-7.0 emissions scenario from 2015 to 2100, with different oceanic and atmospheric initial states for each member. The difference between each member can be used to assess the AR responses to atmospheric or oceanic internal variabilities. In addition, these 40 members are forced by the smoothed CMIP6 biomass burning aerosol forcing.

We also use the pacemaker experiments from CESM2, referred to as PAC. In the PAC simulations, the time-evolving SST anomalies in the eastern tropical Pacific (15°S to 15°N, key region of ENSO) are nudged to the ERSSTv5 during 1880 to 2019, with a 5° latitude buffer region to both hemispheres, and the rest of the model’s coupled climate system is set to freely evolve. In this way, the observed SST variability is imposed in the PAC simulations. Time-varying external, natural, and anthropogenic forcings are specified in these ensembles as CESM2-LE. Since these two sets of experiments, CESM2-LE and PAC, share the same model physics/configurations and external forcings, and the ensemble mean of CESM2-LE represents the model’s forced response to anthropogenic forcing, the comparison of the ensemble means of PAC and CESM2-LE can shed light on the role of eastern tropical Pacific SST variability.

Note that the PAC simulations provide only 850-hPa single-layer winds and specific humidity, so we cannot calculate integrated water vapor transport (IVT) across 300 to 1000 hPa as the AR detection algorithm (*95*) ideally requires. As remedial approach, we use water vapor transport at the single level of 850 hPa (VT850) to approximate the realistic IVT by rescale VT850 to IVT. Given that water vapor transport is strongest at 850 hPa, we believe that this scaling based on VT850 can be used to largely represent changes of 6-hourly IVT. To validate this approach, we calculate the long-term trends of DJF AR frequency in ERA5 using IVT and rescaled VT850 and find no significant differences in their trends and year-to-year variabilities (fig. S12).

### AR detection algorithm

An IVT-based detection algorithm developed by Guan and Waliser (*95*) is used to identify AR on a global scale based on 6-hourly IVT, derived from 6-hourly wind and specific humidity integrated from 1000 to 300 hPa, as $\text{IVT}=\frac{1}{g}\int_{1000}^{300}U\mathrm{qdp}$, where *g* is the gravitational acceleration, ***U*** is the horizontal winds (both zonal and meridional winds), and *q* is the specific humidity.

This AR detection algorithm, an updated and improved version of the algorithm first introduced in Guan and Waliser (*96*), is included in the Atmospheric River Tracking Method Intercomparison Project (ARTMIP) (*97*). It has been widely used in the AR community and is considered a reliable algorithm for detecting ARs on a global scale. The algorithm involves multiple criteria to ensure that the identified ARs are long, narrow, and characterized by concentrated moisture transport: (i) intensity threshold: a location-dependent and monthly dependent 85th percentile of the IVT magnitude or 100 kg m^−1^ s^−1^, whichever is larger, is used as the intensity threshold at each grid to ensure that the selected AR events are significant; (ii) length: the length needs to be longer than 2000 km; and (iii) length-to-width ratio: the length to width is greater than 2. Other criteria, such as the meridional component of mean IVT, the mean transport direction, and coherence, are also applied in the detection algorithm and can be found in Guan and Waliser (*95*). In this study, the monthly AR frequency is calculated by integrating the daily occurrences of ARs within each month, expressed as the number of AR days per month.

To assess the robustness of our results, which rely on the GuanWaliser_v2 algorithm (*95*), we cross-compare trends of DJF AR frequency derived from different algorithms that participated in ARTMIP and two reanalyses (ERA5 and MERRA-2). We use all publicly available AR datasets in ARTMIP for the entire 39-year period (1980 to 2018) to provide a thorough examination of AR frequency trends. Our analysis shows that these trends do not depend on the dataset selected, although the magnitude of trends in MERRA-2 tends to be slightly stronger in the SH (fig. S13). While different algorithms exhibit some regional differences in the AR frequency trends, they generally illustrate a clear poleward shift pattern on a global scale (fig. S14). The Payne (*98*, *99*) and Wille_v2.4 (*100*) algorithms show distinct results as they are designed for regional detection in the Western US and polar regions, respectively. It is worth noting that GuanWaliser_v2 (*95*) and Mundhenk_v3 (*101*) show strong similarity in the poleward shift pattern of AR frequency trends (fig. S14, A and B) because they both use seasonally and spatially varying thresholds. Several ARTMIP studies have shown that algorithms using varying thresholds have better agreement between the reanalysis products and are more suitable for global AR studies than other algorithms that use the fixed threshold (*60*, *102*, *103*). Therefore, we believe that the AR detection algorithm by Guan and Waliser (*95*) is appropriate for this study.

### Eddy-mean flow feedback

Considering the local time rate of change of zonal winds under the zonal average scenario (indicated by the bar operator) and following the approach outlined by Hartmann (*77*)

$$\frac{\partial \bar{u}}{\partial t}=f\bar{v}-\frac{\partial }{\partial y}\left(\bar{u\prime v\prime }\right)-\alpha \bar{u}$$

where $f\bar{v}$ indicates the Coriolis torque term caused by zonal mean meridional winds, $-\frac{\partial }{\partial y}\left(u\prime v\prime \right)$ is the divergence term of eddy momentum flux, measuring how the covariance between zonal and meridional eddy velocities contributes to the redistribution of the zonal momentum in the atmosphere, and $-\alpha \bar{u}$ reflects the frictional drag force with a drag coefficient α. The covariance between zonal and meridional eddy velocities (*u*′*v*′) is calculated following the equation: $u\prime v\prime =\left(u-\bar{u}\right)\left(v-\bar{v}\right)$, where *u* and *v* are daily zonal and meridional winds at each grid point, and $\bar{u}$ and $\bar{v}$ are zonal averages of daily zonal and meridional winds. In this study, we calculate DJF average of eddy momentum flux using daily zonal and meridional winds from ERA5 at various vertical levels.

### Fingerprint pattern matching analysis

To examine the role of internal variability beyond anthropogenic effects on AR activity, 40 simulations from CESM2-LE are used in this study. Given that these members share the same CESM2 model physics and are subjected to the same external and anthropogenic forcing, the spread among 40 members, stemming from slightly different oceanic and atmospheric initial conditions, allows us to assess the impact of non-anthropogenic forcing on AR changes.

To disentangle the relative contributions of internal variability versus anthropogenic forcing in deriving AR activity in the model, we analyze the spatial correlation between the observed 44-year long-term trends in DJF AR frequency and those of each CESM2-LE member, as a criterion to identify two distinct groups within the ensemble: one consists of 6 members with high spatial correlation coefficients (>85th percentile of ascending sorted spatial corrections), indicating a strong agreement with observed trends; another consists of six members with low spatial correction coefficients (<15th percentile), suggesting an inconsistency in CESM2-LE members. This classification is based on a selection criterion of one standard deviation within 40-member ensemble, with six members assigned to each group.

The key to our investigation lies in comparing the trends in Z200 and SST between these two groups. By examining differences in these trends, we aim to discern clear patterns that could indicate the underlying mechanisms driving the disparities in the long-term AR changes between the two groups. Specifically, if the differences between the high-correlation and low-correlation groups reveal well-structured spatial patterns in Z200 or SST trends, it suggests that these variables should play an important role in modulating AR activity. Such insights are crucial for understanding the complex interplay between external forcing and internal variability in shaping the long-term AR changes.

### Statistics analysis

The MCA (*104*), a powerful statistical method based on singular value decomposition, is used extensively in this study to detect dominant covarying patterns between large-scale atmospheric circulation and AR frequency. MCA can isolate pairs of spatial patterns and their corresponding time series by conducting a singular vector decomposition on the temporal covariance matrix of two different fields. This process identifies linear functions of the two variables that exhibit the most pronounced relationship. A key metric in this analysis is the squared covariance fraction, which quantifies the proportion of squared covariance explained by each mode to the total covariance between the two fields. This measure is used to identify the leading and subsequent modes and their significance in overall variability.

In this study, MCA is applied to detrended DJF Z200 and AR frequency on a global scale for the historical period 1979 to 2022. The derived time series from MCA is then correlated with SST to illustrate their relationship. Through these analyses, we aim to understand the coupling between large-scale atmospheric circulation and AR changes, and their relationships with SST, which can provide support to explain the underlying complex mechanisms modulating AR activity.

We use the effective sample size ($N^{*}=N\frac{1-r_{1}r_{2}}{1+r_{1}r_{2}}$; *N* is the total available time steps and *r*_1_ and *r*_2_ are the lag-one autocorrelation coefficients of each variable) to determine the significance of correlations with the confidence level of 95%, as black stippling in most of our figures when the significance of results need to be considered. Linear detrending is used for correlation calculations and MCA to largely remove impacts of anthropogenic forcing on fields of interest, so that the obtained results mainly reflect the connections between the two fields.
