# Supplementary material for: A global poleward shift of atmospheric rivers
Source: Sci Adv. 2024 Oct 11;10(41):eadq0604. doi: 10.1126/sciadv.adq0604 (PMC11468922; doi:10.1126/sciadv.adq0604)
Supplement: Supplementary file 1 — Figs. S1 to S14 [file sciadv.adq0604_sm.pdf]

Science Advances
AAAS

Supplementary Materials for

A global poleward shift of atmospheric rivers

Zhe Li and Qinghua Ding

Corresponding author: Zhe Li, zhe_li@ucsb.edu; Qinghua Ding, qinghua@ucsb.edu

Sci. Adv. **10**, eadq0604 (2024)
DOI: 10.1126/sciadv.adq0604

This PDF file includes:

Figs. S1 to S14

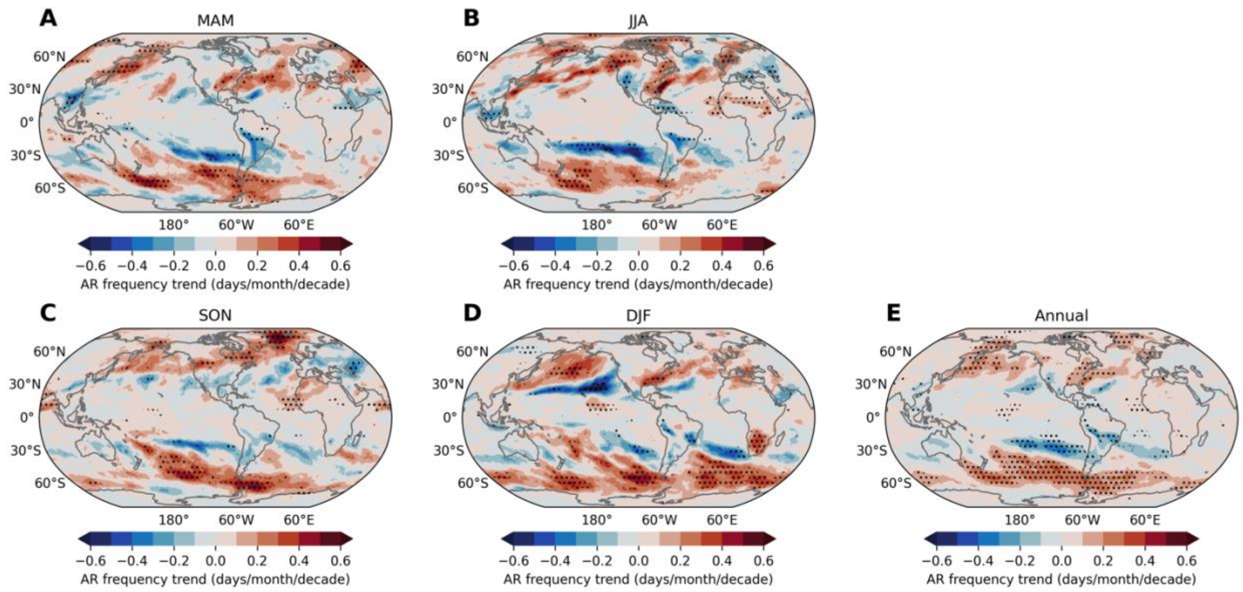

Fig. S1.

Observed seasonal and annual trends of AR frequency. (**A** to **E**) The linear trends of AR frequency from ERA5 for the historical period 1979-2022 across different seasons: boreal Spring (March-April-May, MAM, A), Summer (June-July-August, JJA, B), Fall (September-October-November, SON, C), Winter (December-January-February, DJF, D), and the annual mean (E). Black stippling in all plots indicates statistically significant trends at the 95% confidence level.

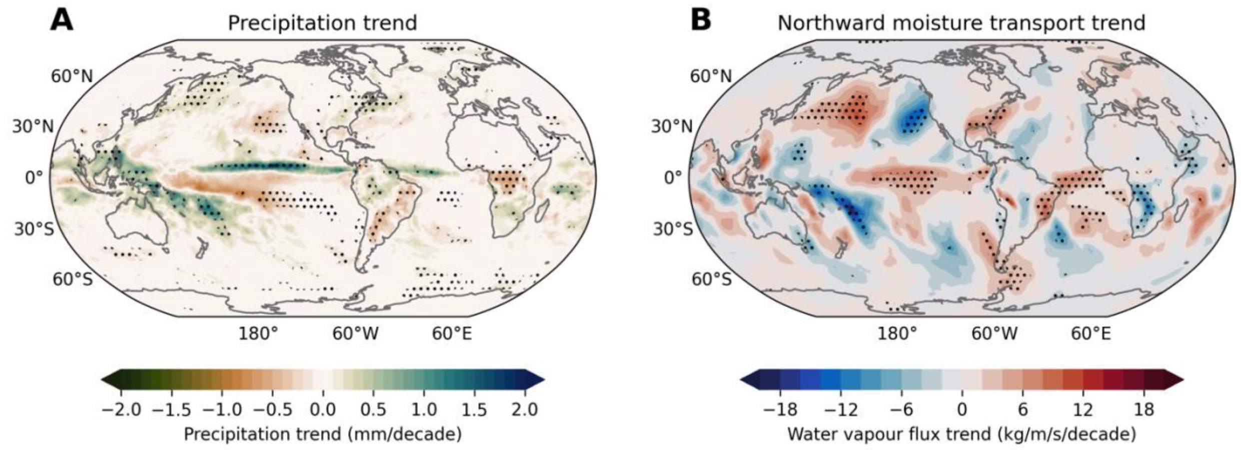

Fig. S2.

Historical trends of precipitation and northward moisture transport in ERA5. (**A** and **B**) The linear trends of DJF total precipitation (A) and northward moisture transport (B) from ERA5 for the historical period 1979-2022. Black stippling in all plots indicates statistically significant trends at the 95% confidence level.

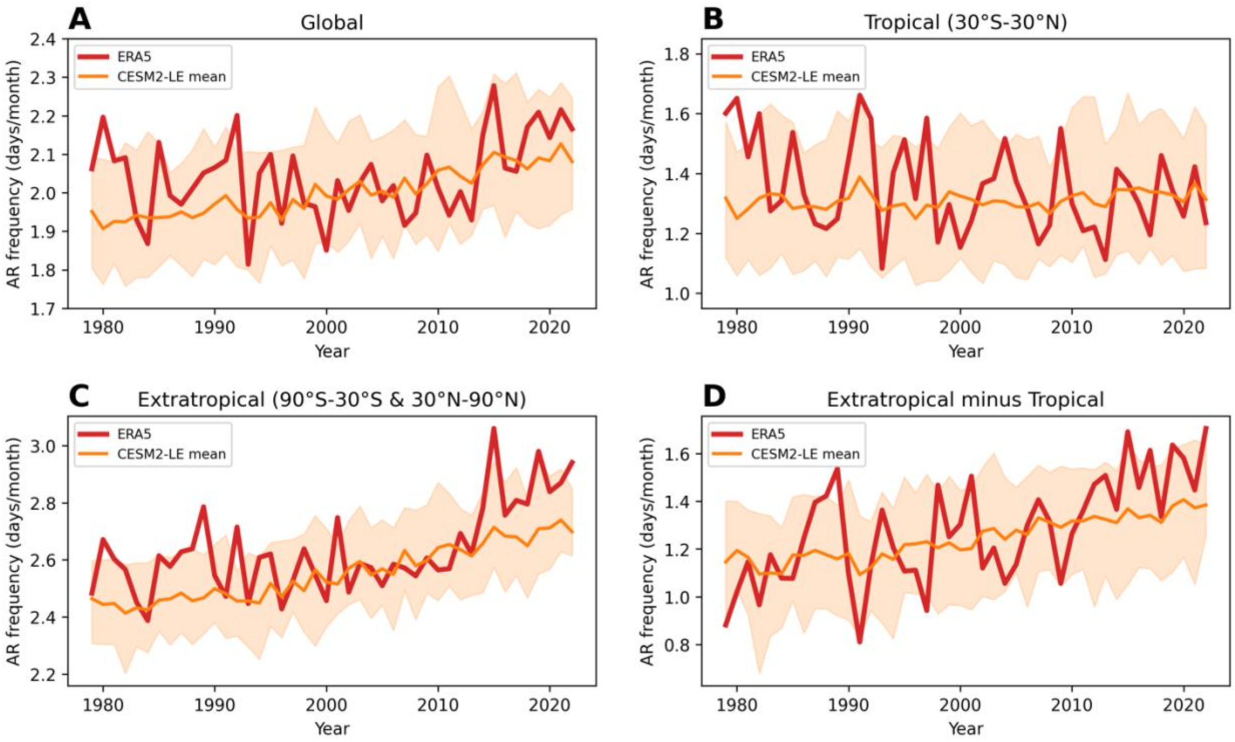

Fig. S3.

Temporal trends of AR frequency in different regions in ERA5 and CESM2-LE. (**A** to **C**) The weighted average of DJF AR frequency from ERA5 (red line) and CESM2-LE (orange line) in the global (A), tropical (30ºS-30ºN, B), and extratropical (90ºS-30ºS & 30ºN-90ºN, C) regions for the historical period 1979-2022. (**D**) The difference between time series of DJF AR frequency weighted average over the extratropical and tropical regions. The orange shadings in the subplots indicate the 5th and 95th percentile of the CESM2-LE members.

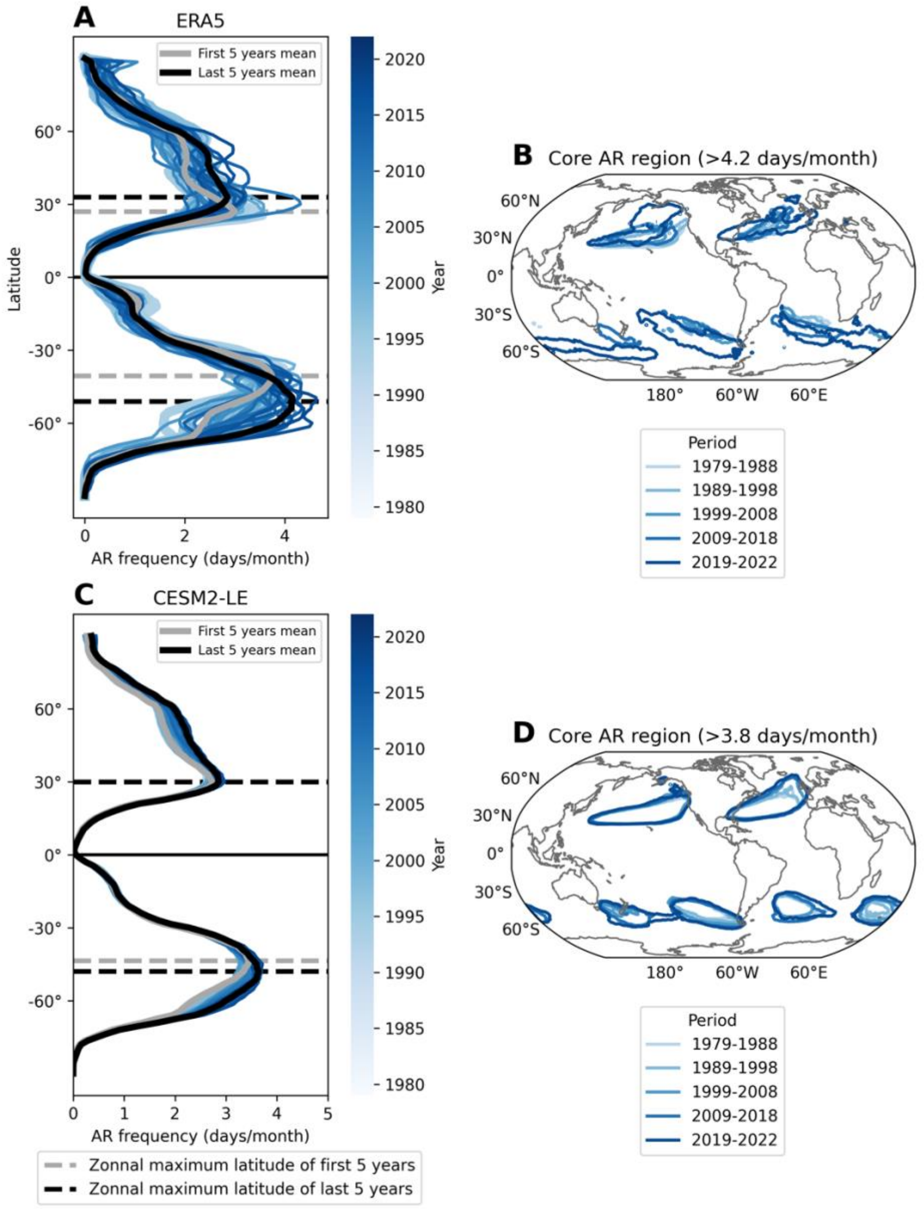

Fig. S4.

Poleward shifts of AR frequency zonal mean and core region in ERA5 and CESM2-LE. (**A** and **C**) The zonal mean of DJF AR frequency from ERA5 (A) and CESM2-LE ensemble mean (C) each year from 1979 to 2022 with lines from light blue to dark blue. The gray and black bolded curves in the subplots are zonal mean of the DJF AR frequency of the first five years 1979-1983 mean and the last five years 2018-2022 mean, respectively. The gray and black horizonal dashed lines indicate the zonal maximum latitudes of the first five years 1979-1983 mean and the last five years 2018-2022 mean, respectively. (**B** and **D**) The core region of DJF AR frequency from ERA5 (B, >4.2 days/month - the threshold defined as the 85th percentile of 44-year DJF AR frequency) and CESM2-LE ensemble mean (D, >3.8 days/month – the threshold adjusted due to lower climatological AR frequency in CESM2-LE) from 1979 to 2022 with 10-year intervals of contours.

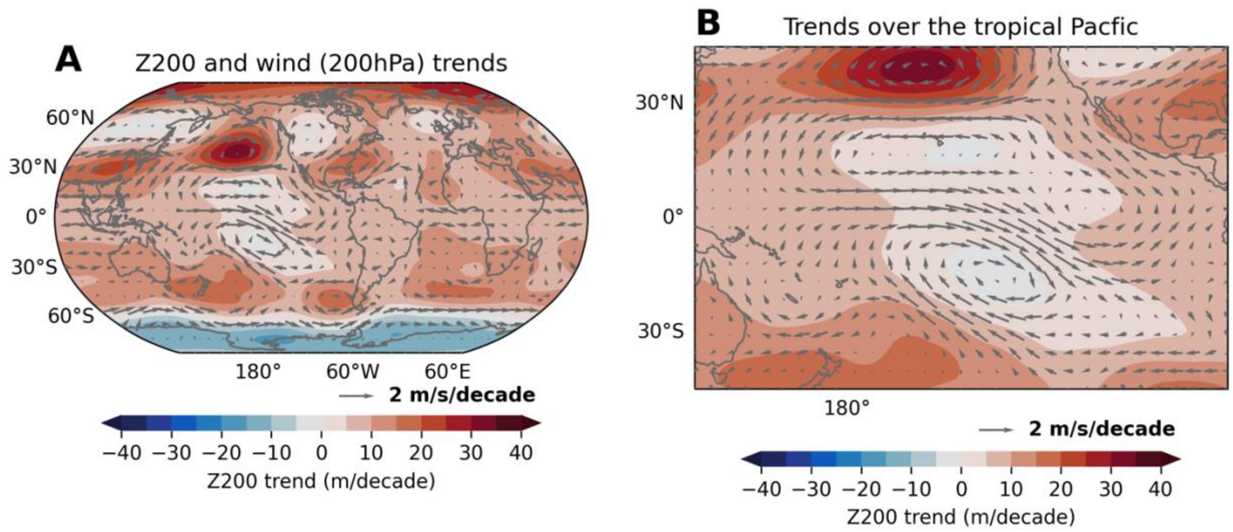

Fig. S5.

Historical trends of Z200 and winds. (**A**) The linear trends of DJF Z200 (shading) and winds at 200 hPa (arrows) for the historical period 1979-2022. (**B**) Same as (A) but zoomed in on the tropical Pacific region.

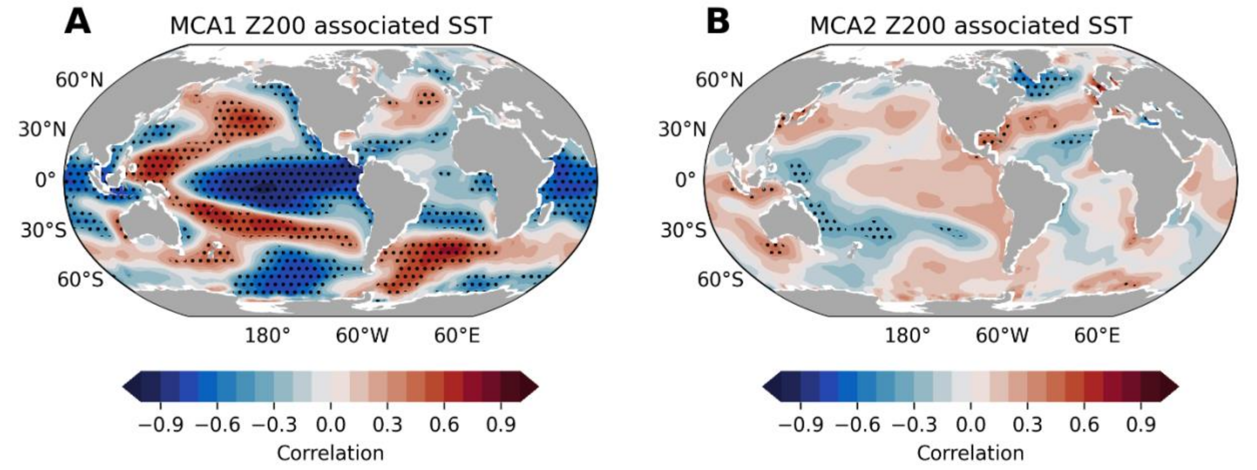

Fig. S6.

MCA Z200 time series associated SST patterns. (**A** and **B**) The correlation of spatial DJF SST with MCA1 Z200 time series (A) and MCA2 Z200 time series (B). All linear trends are removed in calculating correlations. Black stippling in all plots indicates statistically significant correlations at the 95% confidence level.

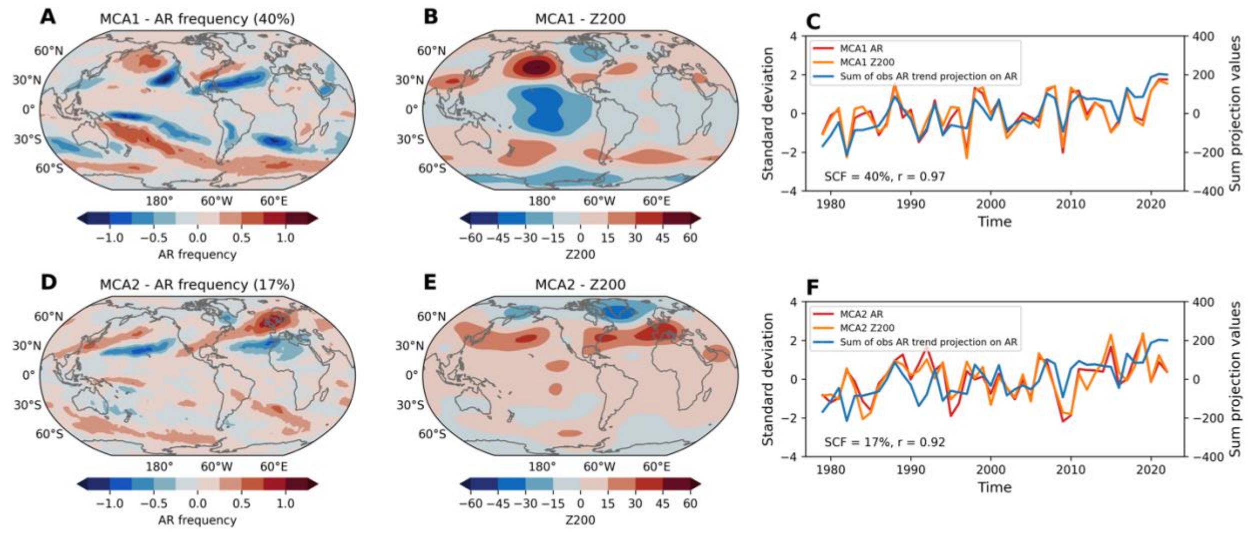

Fig. S7.

Observed statistical relationship between Z200 and AR frequency using MCA (raw, with trend). (**A** to **C**) Results of the leading MCA mode (MCA1) of global AR frequency and Z200 from ERA5 for the historical period 1979-2022 during boreal winter (raw, with trend), with spatial patterns of AR frequency (A) and Z200 (B), and their corresponding standardized time series (C). The time series of the sum of raw AR frequency projection on long-term AR frequency trend from ERA5 is also shown in (C). (**D** to **F**) Same as (A) to (C) but for results of the second MCA mode (MCA2) of two variables. 'SCF' in (C) and (F) indicates the squared covariance fraction of the MCA mode, and 'r' in (C) and (F) indicates the correlation coefficient between the MCA mode time series.

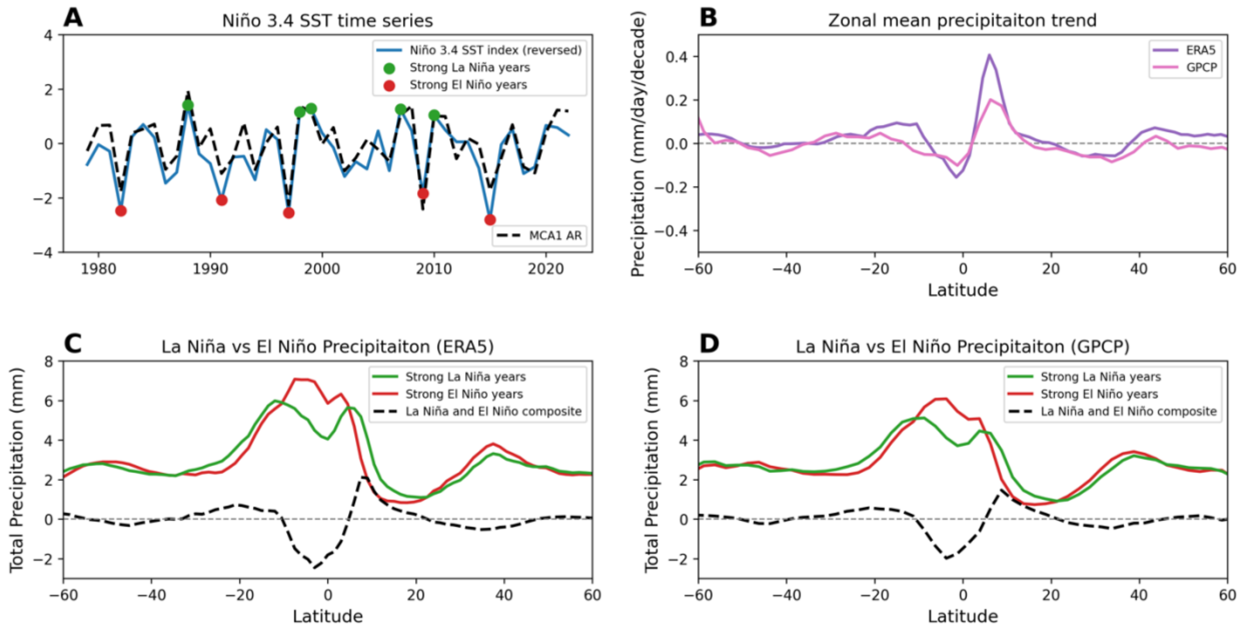

Fig. S8.

Zonal mean total precipitation between La Niña and El Niño winters. (**A**) The time series of revised DJF Niño 3.4 SST index from ERSSTv5 (blue curve) and MCA1 AR (black dashed curve). Five strongest La Niña years and five strongest El Niño years are marked by green and red circles, respectively. (**B**) Zonal mean of DJF precipitation trend for the period 1979-2022 from ERA5 (purple curve) and GPCP (pink curve). (**C**) Zonal mean of DJF ERA5 precipitation averages for five strongest La Niña years (green circles in (A)) and five strongest El Niño years (red circles in (A)) are shown in green and red curves, respectively. (**D**) Same as (C) but from GPCP. The difference of zonal mean DJF precipitation averages between strong La Niña years (green curve) and strong El Niño years (red curve) is also shown in (C) and (D) as the black dashed curve.

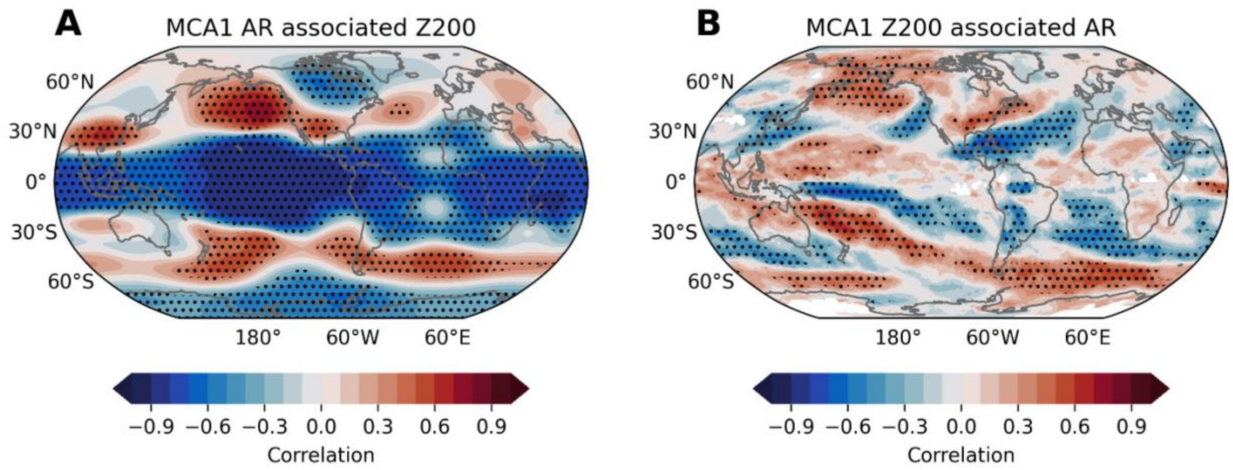

Fig. S9.

MCA1 time series associated Z200 and AR patterns. (**A**) The correlation of MCA1 AR time series with spatial DJF Z200 from ERA5. (**B**) The correlation of MCA1 Z200 time series with spatial DJF SST from ERSSTv5. All linear trends are removed in calculating correlations. Black stippling in all plots indicates statistically significant correlations at the 95% confidence level.

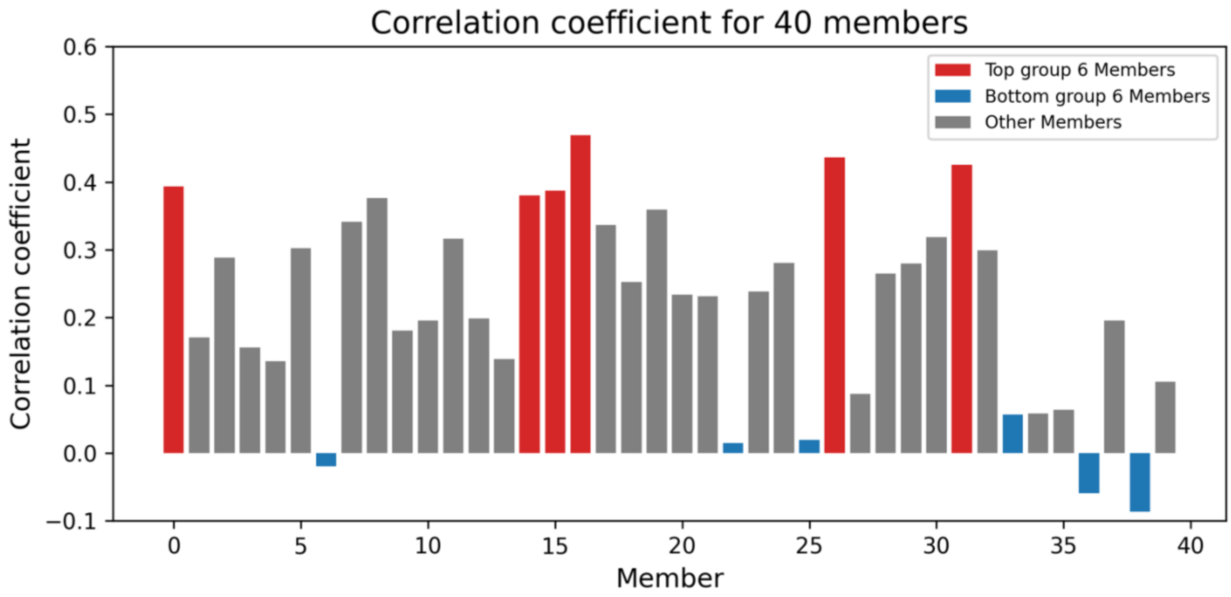

Fig. S10.

A comparison of AR frequency trend patterns between the reanalysis and CESM2-LE members. Spatial correlations between DJF AR frequency trends from ERA5 and CESM2-LE 40 members (each) for the historical period 1979-2022.

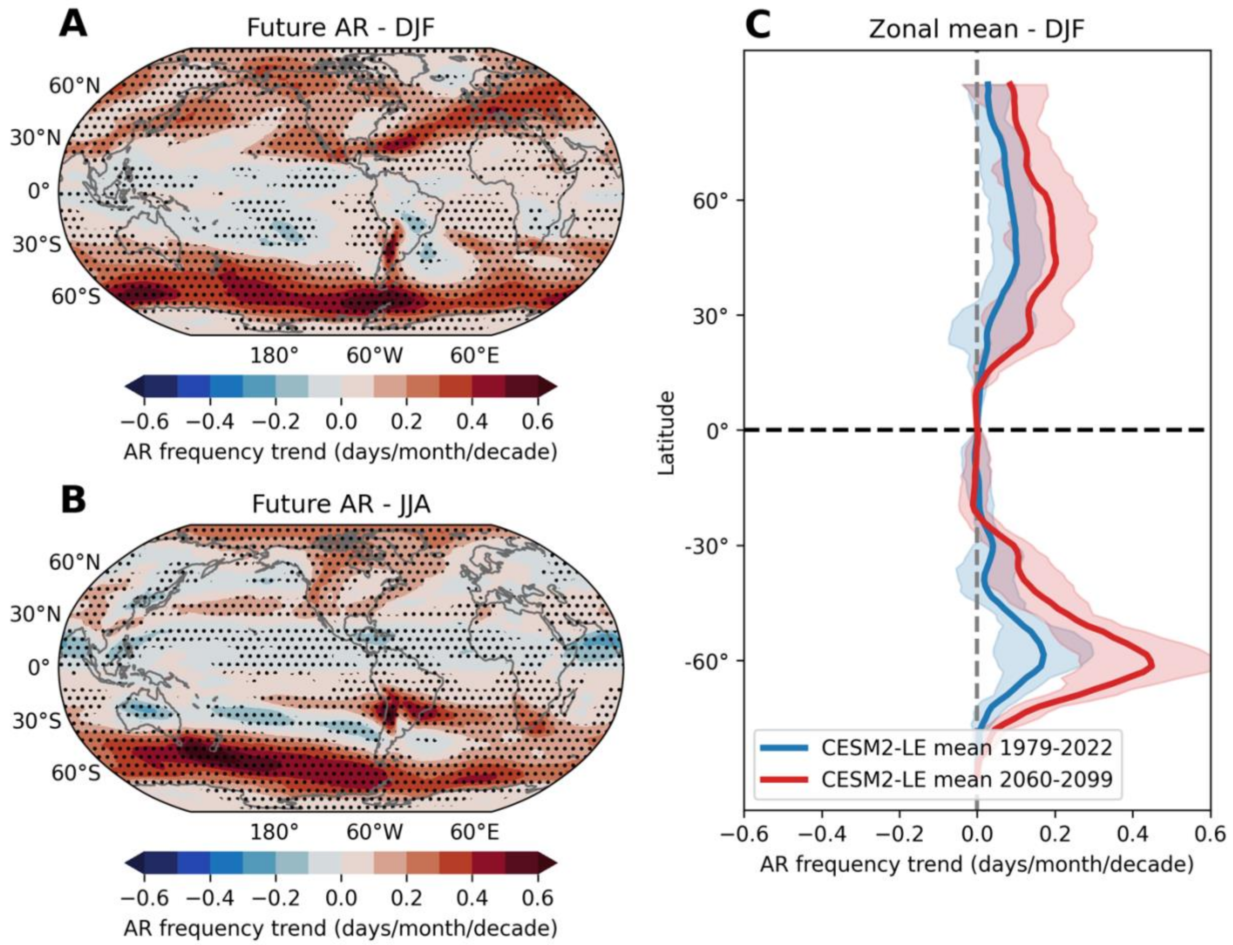

Fig. S11.

Projected future trends of AR frequency in CESM2-LE. (**A** and **B**) The linear trends of AR frequency from CESM2-LE ensemble mean in future simulations (2060-2099) under the SSP3-7.0 scenario in boreal winter (DJF, A) and boreal summer (JJA, B). (**C**) The zonal mean of DJF AR frequency trend in CESM2-LE historical simulations (1979-2022, blue line) and future simulations (2060-2099, red line). The blue and red shadings in (C) indicate the 5th and 95th percentile of the CESM2-LE members in the historical and future simulations. Black stippling in all plots indicates statistically significant trends at the 95% confidence level.

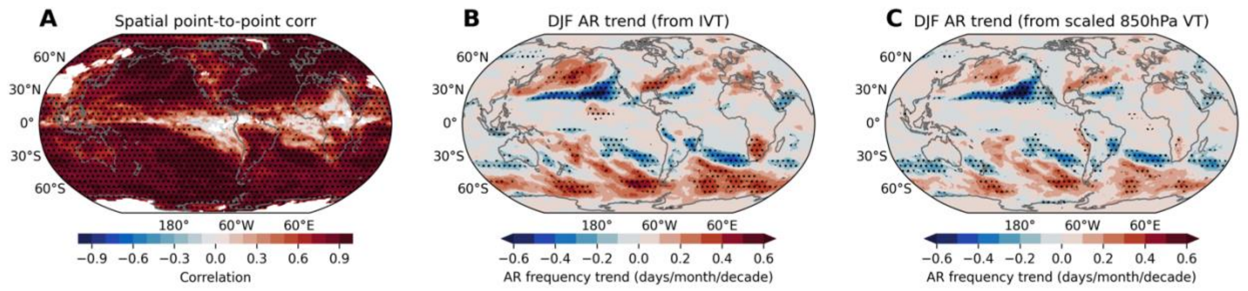

Fig. S12.

A comparison of AR frequency trends using IVT and scaled 850 hPa VT. (**A**) The point-to-point correlation of DJF AR frequency based on 300-1000 hPa integrated water vapor transport (IVT) and scaled 850hPa single-layer water vapor transport (scaled 850hPa VT) in the AR detection algorithm for the historical period 1979-2022. (**B** and **C**) The linear trends of DJF AR frequency from ERA5 for the historical period 1979-2022 based on IVT (B) and scaled 850hPa VT (C) in the AR detection algorithm. Black stippling in all plots indicates statistically significant correlations or trends at the 95% confidence level.

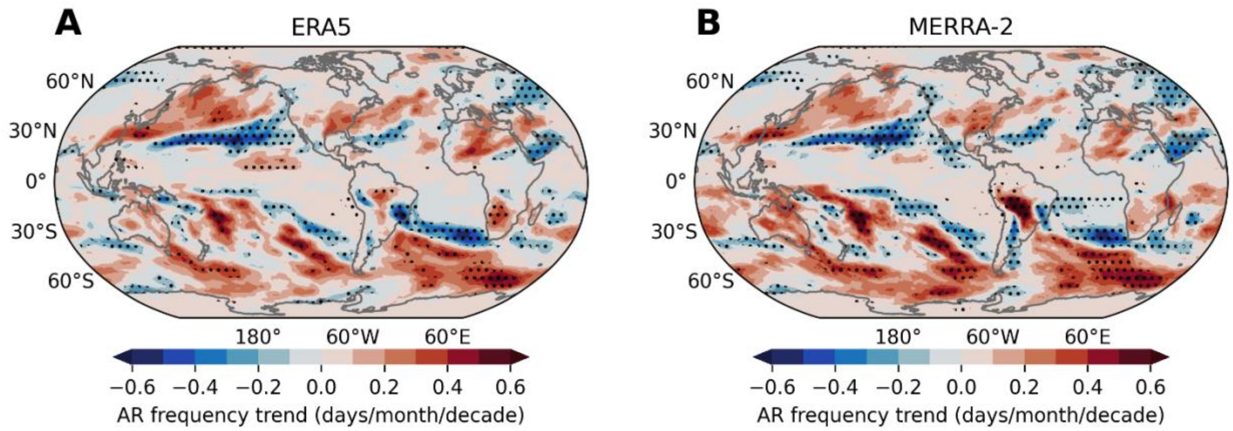

Fig. S13.

A comparison of AR frequency trends using ERA5 and MERRA-2. (**A** and **B**) The linear trends of DJF AR frequency for the historical period 1980-2018 using ERA5 (A) and MERRA-2 (B) in the AR detection algorithm. Black stippling in all plots indicates statistically significant trends at the 95% confidence level. All AR frequency data using in this figure are publicly available in ARTMIP.

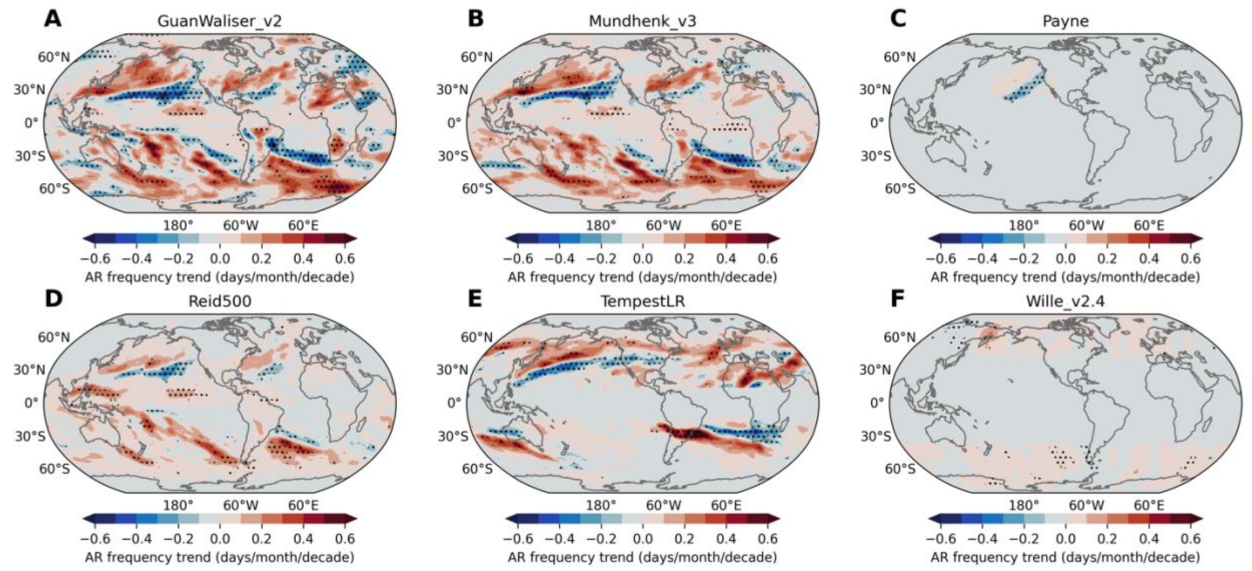

Fig. S14.

A comparison of AR frequency trends in ARTMIP. (**A** to **F**) The linear trends of DJF AR frequency for the historical period 1980-2018 based on AR detection algorithms that participated in ARTMIP using ERA5 data. Black stippling in all plots indicates statistically significant trends at the 95% confidence level. All AR frequency data using in this figure are publicly available in ARTMIP.
